# Perceived Anti-Immigrant Climate, Health Care Discrimination, and Satisfaction with Care Among US Latino Adults

**DOI:** 10.1007/s10903-023-01501-5

**Published:** 2023-05-23

**Authors:** Daniel F. López-Cevallos, Edward D. Vargas, Gabriel R. Sanchez

**Affiliations:** 1 Department of Health Promotion and Policy, School of Public Health and Health Sciences, University of Massachusetts Amherst, 715 North Pleasant St, Amherst, MA 01003 United States; 2 School of Transborder Studies, Arizona State University, 1120 Cady Mall, Tempe, AZ 85281 USA; 3 Department of Political Science, University of New Mexico, 1915 Roma Ave NE, Albuquerque, NM 87106 USA

**Keywords:** Perceived Anti-Immigrant Climate, Health Care Discrimination, Satisfaction with Care, Health Disparities, Latino Populations

## Abstract

A growing body of evidence has documented the effects of discrimination among Latinos. However, little is known about the impacts a noxious sociopolitical climate can have on their health and health care outcomes. The present study explored the associations between perceived anti-immigrant climate, health care discrimination, and satisfaction with care among US Latino adults. We used data from the 2015 Latino National Health and Immigration Survey (n = 1,284), a nationally representative sample of US Latino adults (ages 18 and older). Key predictors included living in a state whose policies are unfavorable towards immigrants, perceived anti-immigrant climate and/or anti-Hispanic climate, and health care discrimination. Ordered logistic regression models evaluated the associations between these predictors (adjusting for other relevant covariates) and satisfaction with care. Latinos living in state that is unfavorable towards immigrants were less likely to be satisfied with medical care they receive. Also, we found that Latinos living in anti-immigrant and anti-Hispanic climates were less likely to be satisfied with care. In both cases, experiencing health care discrimination significantly reduced the odds of satisfaction with care. Latinos’ perception of an anti-immigrant & anti-Hispanic climate and state policies can have detrimental effects on their health and health care outcomes. These results highlight the importance of addressing both community-wide and interpersonal discrimination specific to health care settings, which can have concurrent impacts on the health and well-being of Latino and other minoritized populations.

**Supplementary Information:**

The online version contains supplementary material available at 10.1007/s10903-023-01501-5.

## Introduction

For well over a decade, immigration and health care reform efforts have faced highly contentious and polarizing opinions [1]. Furthermore, from the very moment his candidacy was announced, former President Trump endorsed anti-immigrant and anti-Hispanic/Latino rhetoric and acted on it via federal executive actions (e.g., Title 42, public charge), which has also had ripple effects at the state and local levels [2]. For instance, a 2018 report by the National Conference of State Legislatures showed that immigration legislation increased by 110% in 2017 compared with the previous year [3]. Relatedly, a study found that increases in state Latino populations and state economic stressors associated with the recession led to a greater number of enacted punitive state immigration policies [4].

Such growth in immigration-related state policy, coupled with challenges to the Affordable Care Act (ACA), provide a unique opportunity to assess the effects of anti-immigrant policies. Although there is growing evidence of the impact of racism and discrimination on health care outcomes among minoritized populations [5, 6], little is known about the effects of a perceived anti-immigrant climate on health care outcomes among Latinos.

Recent research found that restrictive federal and state-level immigration policies have negative health effects for Latinos in the U.S., including heightened perceptions of discrimination and deter health insurance enrollment [7, 8]. While previous research has focused primarily on state-level immigration laws, little is known about the impacts a noxious sociopolitical climate can have, and it is unclear how a targeted community’s perceptions of an anti-immigrant climate may impact their health care outcomes. These issues have increased in salience due to the ongoing COVID-19 pandemic. Over the past two and a half years, the pandemic has revealed to the larger public, long-standing health care and social inequities affecting low-income and communities of color (overrepresented among “essential” workforce), including Latinos [9]. Hence, the purpose of the present study is to explore the associations between perceived anti-immigrant climate, health care discrimination, and satisfaction with care among US Latino adults.

## Methods

### Study Design & Participants

We used data from the 2015 Latino National Health and Immigration Survey (LNHIS), a nationally representative survey intended for examining the relationship between immigrant policy and Latino health and well-being. The 2015 LNHIS surveyed 1,493 participants, consisting of a mix of cell phone and landline households (n = 989), and web-based surveys (n-504). Our analytical sample (n = 1,284) was limited to participants who answered all questions included in the present study.

The polling firm Latino Decisions designed and implemented the survey. They selected 44 states (and Puerto Rico) with the largest number of Latino residents for the sampling design, accounting for 91% of the U.S. Latino adult population. Both phone (cell – 35%; landline – 65%, interviewer-administered) and web respondents (self-administered) could be interviewed in either English or Spanish, per their preference. The final dataset was weighted to the 2013 Current Population Survey universe estimate of Latino adults by age, place of birth, gender, and state. The survey lasted for 28 min, on average, and was conducted between January and March 2015 (response rate – 18%). The research protocol was approved by the institutional review board (IRB) of The University of New Mexico.

### Measures

The outcome variable, satisfaction with care, asked respondents: ““Now thinking about the medical care you received in the past year, how satisfied are you with the quality of medical care available to you and your family (very satisfied, satisfied, dissatisfied (or) very dissatisfied).“ We used the Behavioral Model for Vulnerable Populations (BMVP) as our guiding conceptual framework [10]. The BMVP considers satisfaction with care as a function of predisposing (e.g., demographics), enabling (e.g., experiences of discrimination), and need (e.g., health status) factors. Hence, our study’s key predictors were: 1) living in a state whose policies are unfavorable towards immigrants (Thinking about the immigrant population in your state, would you describe [STATE] policies as favorable or unfavorable towards immigrants?), and 2) perceived anti-immigrant and/or anti-Hispanic climate (Some people have said that there seems to be a lot of anti-immigrant, and even anti- Hispanic, statements, policies, and attitudes surfacing in recent years; while other people have said that no such anti-immigrant environment exists today. How do you feel? Do you feel there is definitely an anti-immigrant or anti-Hispanic environment today, there is somewhat of an anti-immigrant or anti-Hispanic environment, or, do you think no such environment exists today?). Other relevant covariates included: experiencing health care discrimination (Have you ever been treated unfairly at a doctor office, clinic, or hospital?), household primary care visits in past year, immigration generation, age, sex, educational level, income, health insurance, marital status, country of origin, skin color, and language of survey.

### Data Analysis

Summary statistics were calculated for all study variables. Ordered logistic regression models evaluated the associations between living in a state whose policies are unfavorable towards immigrants, perceived anti-immigrant/anti-Hispanic climate, and satisfaction with care. We further explored interactions between key predictors and health insurance status as a robustness check (see supplemental file 1). All analyses used state fixed effects to account for unobserved state-level factors such as access to care, local immigration policies, and state economy. We utilized Stata 14 (College Station, TX).

## Results

On average, Latino respondents reported being somewhat satisfied with the quality of their health care and 39% reported their state as unfavorable towards immigrants (Table 1). The mean number of respondents who reported that there is an anti-immigrant climate is 24%, anti-Hispanic sentiment is 16%, both anti-immigrant and anti-Hispanic was 39% and those who responded neither anti-Hispanic nor anti-immigrant was 21%. The descriptive results also show that 10% of respondents reported being discriminated against while receiving medical care and on average respondents had almost 3 visits to primary care doctors or clinics in the last 12 months.

**Table 1:** Weighted summary statistics using the 2015 national latino health and immigration survey. (n = 1,284)

Variables	Mean	Std. Dev.	Min	Max
Satisfaction with care^a^	4.12	1.08	1	5
Unfavorable State Immigrant Policy	0.39	--	0	1
Both Anti-Immigrant/Hispanic	0.39	--	0	1
Anti-Hispanic Climate	0.16	--	0	1
Anti-Immigrant Climate	0.24	--	0	1
No Anti-Immigrant/Hispanic Climate	0.21	--	0	1
Health Care Discrimination	0.10	--	0	1
Generational Status^b^	1.65	0.76	1	3
First Generation	0.52	--	0	1
Second Generation	0.30	--	0	1
Third Generation and Above	0.18	--	0	1
Female	0.54	--	0	1
Education^c^	5.38	2.21	1	10
Age	41.16	15.72	18	98
Income Missing	0.21	--	0	1
Income: Less than 20k	0.18	--	0	1
Income: 20-39 K	0.22	--	0	1
Income: 40k-60k	0.14	--	0	1
Income: 60k -80k	0.09	--	0	1
Income: 80k-100k	0.06	--	0	1
Income: 100k-150k	0.07	--	0	1
Income: 150k+	0.03	--	0	1
Currently Uninsured	0.19	--	0	1
Married^d^	0.53	--	0	1
Spanish^e^	0.42	--	0	1
Mexican Origin^f^	0.57	--	0	1
Skin Color^g^	2.55	0.99	1	5
Household primary care visits	2.73	4.21	0	87

^a^ Satisfaction with care: (1 = Very dissatisfied-5%, 2 = Somewhat dissatisfied-5%, 3 = Neither satisfied nor dissatisfied-9%, 4 = Somewhat satisfied-35%, 5 = Very satisfied-46%)

^b^ Generational Status (1 = First Generation, 2 = Second Generation, 3-Third Generation and above)

^c^ Highest education levels completed: (1 = Grade 1–8, 2 = Some HS, 3 = HS, 4 = Some College, 5 = College Grad,

6 = Post-Grad) .

^d^ Marital Status (0 = Single, Divorced, Widowed, Other, 1 = Married).

^e^Spanish: (0 = English, 1 = Spanish).

^f^ Mexican Origin: (0 = All Else, 1 = Mexican).

^g^ Skin Color: (1 = Very Light, 2 = Light, 3 = Medium, 4 = Dark, 5 = Very Dark).

Multivariable analyses (Table 2) show that Latinos living in state that is unfavorable towards immigrants were less likely to be satisfied with care they received (OR: 0.69, 95% CI: 0.54–0.87). When examining the overall climate, we found that Latinos living in both anti-immigrant and anti-Hispanic climates were less likely to be satisfied with care (OR: 0.64, 95% CI: 0.47–0.87). In both cases, Latinos who experienced health care discrimination were also less likely to be satisfied with their care (OR: 0.58, 95% CI: 0.39–0.84 for unfavorable state immigrant policy; and OR: 0.62, 95% CI: 0.42–0.89 for anti-immigrant or anti-Hispanic climate).

**Table 2:** Ordered logistic regression results of the association between perceived anti-immigrant climate, health care discrimination, and satisfaction with care among US Latino adults

Variables	β	Odds Ratio	β	Odds Ratio
Unfavorable State Immigrant Policy	-0.373***	0.688***		
Reference Category: No Anti-Immigrant or Anti-Hispanic Climate				
Anti-Immigrant Climate			-0.026	0.975
Anti-Hispanic Climate			-0.137	0.872
Both Anti-Immigrant/Hispanic			-0.452***	0.637***
Health Care Discrimination	-0.553***	0.575***	-0.482**	0.617**
Reference Category: 1st Generation				
2nd Generation	0.011	1.011	-0.026	0.974
3rd Generation	0.200	1.221	0.134	1.144
Female	0.060	1.061	0.043	1.044
Education	-0.052	0.949	-0.066*	0.936*
Age	0.009**	1.009**	0.009**	1.009**
Reference Income: Less than 20k				
Income Missing	-0.014	0.986	-0.116	0.891
Income: 20-39 K	-0.005	0.995	-0.103	0.902
Income: 40k-60k	-0.390*	0.677*	-0.459**	0.632**
Income: 60k -80k	-0.219	0.803	-0.308	0.735
Income: 80k-100k	0.161	1.174	0.170	1.185
Income: 100k-150k	0.184	1.202	0.055	1.057
Income: 150k+	0.610*	1.840*	0.577	1.780
Currently Uninsured	-0.905***	0.405***	-1.010***	0.364***
Married	-0.012	0.988	-0.048	0.953
Spanish	0.312**	1.365**	0.235	1.265
Mexican Origin	-0.010	0.990	0.037	1.037
Skin Color	-0.006	0.994	-0.022	0.978
Household primary care visits	0.036**	1.036**	0.018	1.018
Adjusted R-squared	0.0600		0.0593	

Lastly, we explored interactions between health insurance status and key predictors (living in a state whose policies are unfavorable towards immigrants; perceived anti-immigrant/anti-Hispanic climate; health care discrimination). Results (available as a supplemental file 1) show no difference by health insurance status on the relationship between living in a state whose policies are unfavorable towards immigrants; perceived anti-immigrant/anti-Hispanic climate, or health care discrimination, and satisfaction with medical care.

## Discussion

This study is the first, to our knowledge, to examine perceptions of anti-immigrant climate, health care discrimination, and satisfaction with care, among a nationally representative sample of US Latino adults. Our findings suggest that, even after accounting for perceived discrimination in health care settings, Latinos’ perception of an anti-immigrant & anti-Hispanic climate and state policies (broadly speaking) is associated with lower satisfaction of their health care experiences. In that sense, our findings support previous research documenting the impact of systemic racism and discrimination on the health and health care outcomes of U.S. racial/ethnic minority populations [5], and contribute to the limited but growing body of research among Latino populations [8]. Since the survey was conducted right before the COVID-19 pandemic, our study provides insights into the state of perceived discrimination (both at the community and health care setting levels) that may help explain the challenges public health institutions continue to have in reaching out to Latino communities (mistrustful of institutions), leading to disproportionately poorer health outcomes [11]. Interestingly, it was the combined anti-immigrant and anti-Hispanic climate that significantly reduced satisfaction with care. Such effect may be explained in part by the conflation of immigrant and Hispanic/Latino communities as one and the same in immigration rhetoric and policy debates [1, 2, 6].

Our study had several limitations. First, we are constrained in our ability to establish causal relationships given the cross-sectional nature of our study. However, to date there are no other available datasets that have explore issues regarding anti-immigrant policies and overall climate over time. The 2015 LNHIS is the first nationally representative survey to explore Latinos’ health, experiences with the changing healthcare marketplace, and outlook on immigration and racial issues. However, no such survey has been conducted since then, at a time when much has been discussed and acted upon at the federal, state, and local levels. For instance, the Trump administration utilized a public health emergency mechanism (i.e., title 42) during the COVID-19 pandemic as an immigration enforcement mechanism in pursuit of an anti-immigrant/anti-Latino agenda, which may have ongoing implications for Latinos health care satisfaction for years to come [2].

Second, our study relies on self-report which may be prone to respondent bias and does not incorporate measures of enacted policies to contrast such perceptions. Future research may consider incorporating other policy measures (both related to immigration and health care policy) and monitoring its effects over time, given the likelihood of continued policy change in both areas, as well as triangulating community perceptions with state-specific data.

These results highlight the importance of addressing both community-wide, and interpersonal discrimination specific to health care settings, which can have concurrent impacts on the health and well-being of Latino and other minoritized populations. Future studies are needed to further characterize and monitor the longitudinal effects of perceptions and enacted policies on health and health care outcomes among Latinos, the largest racial/ethnic minority in the US. Moreover, the role of federal policies (e.g., Title 42, changes in asylum seeking procedures, public charge) and how they are being framed in the media merit further research. It would be interesting to explore how perception of these policies and media portrays affect health and health care outcomes in this population. The importance of our findings is magnified considering the COVID-19 pandemic. Historic and contemporary social and structural conditions have led to deepening inequalities for Latinos (overrepresented among “essential workers”) in COVID-19 cases, hospitalizations and deaths [11]. Our findings suggest that the devastating effects of the pandemic on the Latino community were driven at least in part, by a prevalent anti-immigrant socio-political climate. Unless we take steps to address both interpersonal and systemic racism, Latinos will unfortunately continue to face COVID and other health issues in an ongoing context of discrimination (both within and outside health care settings).

### Electronic supplementary material

Supplementary File 1
