# Supplementary material for: Perceived Anti-Immigrant Climate, Health Care Discrimination, and Satisfaction with Care Among US Latino Adults
Source: J Immigr Minor Health. 2023 May 23;25(5):1197–201. doi: 10.1007/s10903-023-01501-5 (PMC10203659; doi:10.1007/s10903-023-01501-5)
Supplement: Supplementary file 1 [file 10903_2023_1501_MOESM1_ESM.docx]

***Supplemental File***

**Table S1** Ordered logistic regression results of perceived anti-immigrant climate, health care discrimination, and satisfaction with care among US Latino adults, stratified by health insurance status

|  | Insured | | Uninsured | | Insured | | Uninsured | |
| --- | --- | --- | --- | --- | --- | --- | --- | --- |
| Variables | β | Odds Ratios | β | Odds Ratios | β | Odds Ratios | β | Odds Ratios |
| Unfavorable State Immigrant Policy | -0.367*** | 0.693*** | -0.533* | 0.587* |  |  |  |  |
| Reference Category: No Anti-Immigrant or Anti-Hispanic Climate | | |  |  |  |  |  |  |
| Anti-Immigrant Climate |  |  |  |  | 0.068 | 1.070 | 0.091 | 1.095 |
| Anti-Hispanic Climate |  |  |  |  | 0.010 | 1.010 | -0.733 | 0.481 |
| Both Anti-Immigrant/Hispanic |  |  |  |  | -0.399** | 0.671** | -0.528 | 0.590 |
| Health Care Discrimination | -0.487** | 0.614** | -1.057** | 0.348** | -0.461** | 0.631** | -0.794* | 0.452* |
| Reference Category: 1st Generation | |  |  |  |  |  |  |  |
| 2nd Generation | -0.164 | 0.848 | 0.461 | 1.585 | -0.212 | 0.809 | 0.472 | 1.603 |
| 3rd Generation | 0.164 | 1.178 | 0.404 | 1.498 | 0.100 | 1.106 | 0.053 | 1.055 |
| Female | -0.021 | 0.980 | 0.689** | 1.992** | -0.059 | 0.943 | 0.787** | 2.198** |
| Education | -0.056 | 0.945 | -0.079 | 0.924 | -0.062* | 0.940* | -0.125 | 0.883 |
| Age | 0.014*** | 1.014*** | -0.024* | 0.976* | 0.012*** | 1.013*** | -0.022 | 0.979 |
| Reference Income: Less than 20k |  |  |  |  |  |  |  |  |
| Income Missing | 0.168 | 1.183 | -0.627 | 0.534 | 0.124 | 1.132 | -0.812* | 0.444* |
| Income: 20K-39K | -0.250 | 0.779 | 0.821** | 2.274** | -0.359* | 0.698* | 0.781** | 2.183** |
| Income: 40k-60k | -0.454* | 0.635* | -0.203 | 0.816 | -0.532** | 0.587** | -0.340 | 0.712 |
| Income: 60k -80k | -0.369 | 0.691 | 1.087 | 2.966 | -0.467* | 0.627* | 1.200* | 3.322* |
| Income: 80k-100k | 0.257 | 1.293 | -0.726 | 0.484 | 0.289 | 1.335 | -0.626 | 0.535 |
| Income: 100k-150k | 0.382 | 1.465 | -2.090* | 0.124* | 0.246 | 1.279 | -2.189** | 0.112** |
| Income: 150k+ | 0.730* | 2.076* | 0.341 | 1.406 | 0.704* | 2.022* | 0.747 | 2.111 |
| Married | -0.254* | 0.776* | 0.470 | 1.601 | -0.263* | 0.769* | 0.413 | 1.512 |
| Spanish | 0.211 | 1.236 | 0.723 | 2.061 | 0.216 | 1.241 | 0.396 | 1.485 |
| Mexican Origin | 0.109 | 1.115 | -0.037 | 0.963 | 0.151 | 1.163 | -0.185 | 0.831 |
| Skin Color | 0.029 | 1.029 | 0.096 | 1.101 | -0.014 | 0.986 | 0.154 | 1.167 |
| Household primary care visits | 0.024 | 1.025 | 0.130 | 1.139 | 0.017 | 1.017 | -0.082 | 0.921 |
| Adjusted R-squared | 0.0537 |  | 0.161 |  | 0.0548 |  | 0.159 |  |

*Notes: *** p<0.01, ** p<0.05, * p<0.1, State fixed effects using complex survey weights.*

**Table S2** Ordered logistic regression interaction results of living in an unfavorable immigrant state and satisfaction with care among US Latino adults

|  | Interaction of Unfavorable and Uninsured | | Interaction of Discrimination and Uninsured | |
| --- | --- | --- | --- | --- |
| Variables | β | Odds Ratios | β | Odds Ratios |
|  |  |  |  |  |
| Unfavorable State Immigrant Policy | -0.379*** | 0.684*** | -0.372*** | 0.689*** |
| Currently Uninsured | -0.918*** | 0.399*** | -0.879*** | 0.415*** |
| Favorable x Insured | 0.000 | 1.000 |  |  |
| Favorable x Uninsured | 0.000 | 1.000 |  |  |
| Unfavorable x Insured | 0.000 | 1.000 |  |  |
| Unfavorable x Uninsured | 0.030 | 1.030 |  |  |
| Health Care Discrimination | -0.553*** | 0.575*** | -0.498** | 0.608** |
| No Discrimination x Insured |  |  | 0.000 | 1.000 |
| No Discrimination x Uninsured |  |  | 0.000 | 1.000 |
| Discrimination x Insured |  |  | 0.000 | 1.000 |
| Discrimination x Uninsured |  |  | -0.237 | 0.789 |
| Reference Category: 1st Generation |  |  |  |  |
| 2nd Generation | 0.011 | 1.011 | 0.012 | 1.013 |
| 3rd Generation | 0.199 | 1.221 | 0.204 | 1.226 |
| Female | 0.059 | 1.061 | 0.062 | 1.064 |
| Education | -0.053 | 0.949 | -0.052 | 0.949 |
| Age | 0.009** | 1.009** | 0.009** | 1.009** |
| Reference Income: Less than 20k |  |  |  |  |
| Income Missing | -0.013 | 0.987 | -0.015 | 0.985 |
| Income: 20K-39K | -0.004 | 0.996 | -0.007 | 0.994 |
| Income: 40k-60k | -0.389* | 0.678* | -0.390* | 0.677* |
| Income: 60k -80k | -0.218 | 0.804 | -0.224 | 0.799 |
| Income: 80k-100k | 0.161 | 1.175 | 0.160 | 1.174 |
| Income: 100k-150k | 0.183 | 1.201 | 0.183 | 1.200 |
| Income: 150k+ | 0.611* | 1.842* | 0.605* | 1.831* |
| Married | -0.013 | 0.987 | -0.014 | 0.986 |
| Spanish | 0.311** | 1.365** | 0.316** | 1.372** |
| Mexican Origin | -0.011 | 0.989 | -0.011 | 0.989 |
| Skin Color | -0.006 | 0.994 | -0.007 | 0.993 |
| Household primary care visits | 0.036** | 1.036** | 0.036** | 1.037** |
| Adjusted R-squared | 0.0600 |  | 0.0601 |  |

*Notes: *** p<0.01, ** p<0.05, * p<0.1, State fixed effects using complex survey weights.*

**Table S3** Ordered logistic regression interaction results of living in an anti-immigrant and anti-Hispanic state and satisfaction with care among US Latino adults

|  | Interaction of Anti-Immigrant Climate and Uninsured | | Interaction of Discrimination and Uninsured | |
| --- | --- | --- | --- | --- |
| Variables | β | Odds Ratios | β | Odds Ratios |
| Reference Category: No Anti-Immigrant or Anti-Hispanic Climate | |  |  |  |
| Anti-Immigrant Climate | -0.030 | 0.970 | -0.030 | 0.970 |
| Anti-Hispanic Climate | -0.017 | 0.983 | -0.136 | 0.873 |
| Both Anti-Immigrant/Hispanic | -0.423** | 0.655** | -0.452*** | 0.636*** |
| Currently Uninsured | -0.810** | 0.445** | -0.987*** | 0.373*** |
| No Anti-Immigrant or Anti-Hispanic Climate x Insured | 0.000 | 1.000 |  |  |
| No Anti-Immigrant or Anti-Hispanic Climate x Uninsured | 0.000 | 1.000 |  |  |
| Anti-Immigrant Climate x Insured | 0.000 | 1.000 |  |  |
| Anti-Immigrant Climate x Uninsured | -0.025 | 0.976 |  |  |
| Anti-Hispanic Climate x Insured | 0.000 | 1.000 |  |  |
| Anti-Hispanic Climate x Uninsured | -0.585 | 0.557 |  |  |
| Both Anti-Immigrant/Hispanic x Insured | 0.000 | 1.000 |  |  |
| Both Anti-Immigrant/Hispanic x Uninsured | -0.198 | 0.820 |  |  |
| Health Care Discrimination | -0.473** | 0.623** | -0.435** | 0.647** |
| No Discrimination x Insured |  |  | 0.000 | 1.000 |
| No Discrimination x Uninsured |  |  | 0.000 | 1.000 |
| Discrimination x Insured |  |  | 0.000 | 1.000 |
| Discrimination x Uninsured |  |  | -0.203 | 0.816 |
| Reference Category: 1st Generation |  |  |  |  |
| 2nd Generation | -0.025 | 0.975 | -0.024 | 0.977 |
| 3rd Generation | 0.143 | 1.154 | 0.138 | 1.148 |
| Female | 0.043 | 1.044 | 0.045 | 1.046 |
| Education | -0.067** | 0.936** | -0.066* | 0.936* |
| Age | 0.009** | 1.009** | 0.009** | 1.009** |
| Reference Income: Less than 20k |  |  |  |  |
| Income Missing | -0.115 | 0.891 | -0.118 | 0.889 |
| Income: 20K-39K | -0.104 | 0.901 | -0.104 | 0.901 |
| Income: 40k-60k | -0.463** | 0.630** | -0.460** | 0.631** |
| Income: 60k -80k | -0.305 | 0.737 | -0.313 | 0.731 |
| Income: 80k-100k | 0.184 | 1.202 | 0.169 | 1.184 |
| Income: 100k-150k | 0.051 | 1.052 | 0.053 | 1.054 |
| Income: 150k+ | 0.586 | 1.797 | 0.571 | 1.771 |
| Married | -0.047 | 0.954 | -0.049 | 0.952 |
| Spanish | 0.232 | 1.262 | 0.238 | 1.269 |
| Mexican Origin | 0.025 | 1.025 | 0.035 | 1.036 |
| Skin Color | -0.016 | 0.984 | -0.023 | 0.977 |
| Household primary care visits | 0.017 | 1.017 | 0.018 | 1.019 |
| Adjusted R-squared | 0.0599 |  | 0.0594 |  |

*Notes: *** p<0.01, ** p<0.05, * p<0.1, State fixed effects using complex survey weights..*

**Table S4** Ordered logistic regression results of the association between perceived anti-immigrant climate, health care discrimination, and satisfaction with care among US Latino adults

| VARIABLES | β | Odds Ratio | β | Odds Ratio | β | Odds Ratio | β | Odds Ratio |
| --- | --- | --- | --- | --- | --- | --- | --- | --- |
| Unfavorable State Immigrant Policy | -0.372*** | 0.689*** | -0.373*** | 0.688*** |  |  |  |  |
| Reference Category: No Anti-Immigrant or Anti-Hispanic Climate | | | | | |  |  |  |
| Anti-Immigrant Climate | | |  |  | -0.088 | 0.916 | -0.026 | 0.975 |
| Anti-Hispanic Climate | |  |  |  | -0.178 | 0.837 | -0.137 | 0.872 |
| Both Anti-Immigrant/Hispanic | | |  |  | -0.476*** | 0.621*** | -0.452*** | 0.637*** |
| Health Care Discrimination | | | -0.553*** | 0.575*** |  |  | -0.482** | 0.617** |
| Reference Category: 1st Generation | | | | | | |  |  |
| 2nd Generation | -0.030 | 0.970 | 0.011 | 1.011 | -0.067 | 0.935 | -0.026 | 0.974 |
| 3rd Generation | 0.186 | 1.205 | 0.2 | 1.221 | 0.111 | 1.118 | 0.134 | 1.144 |
| Female | 0.040 | 1.041 | 0.06 | 1.061 | 0.033 | 1.033 | 0.043 | 1.044 |
| Education | -0.051 | 0.950 | -0.052 | 0.949 | -0.064* | 0.938* | -0.066* | 0.936* |
| Age | 0.009** | 1.009** | 0.009** | 1.009** | 0.009** | 1.009** | 0.009** | 1.009** |
| Reference Income: Less than 20k | | | | | | | |  |
| Income Missing | 0.008 | 1.009 | -0.014 | 0.986 | -0.112 | 0.894 | -0.116 | 0.891 |
| Income: 20K-39K | 0.025 | 1.025 | -0.005 | 0.995 | -0.076 | 0.927 | -0.103 | 0.902 |
| Income: 40k-60k | -0.354* | 0.702* | -0.390* | 0.677* | -0.433** | 0.649** | -0.459** | 0.632** |
| Income: 60k -80k | -0.208 | 0.813 | -0.219 | 0.803 | -0.278 | 0.757 | -0.308 | 0.735 |
| Income: 80k-100k | 0.182 | 1.200 | 0.161 | 1.174 | 0.187 | 1.206 | 0.17 | 1.185 |
| Income: 100k-150k | 0.220 | 1.246 | 0.184 | 1.202 | 0.081 | 1.085 | 0.055 | 1.057 |
| Income: 150k+ | 0.622* | 1.863* | 0.610* | 1.840* | 0.594 | 1.812 | 0.577 | 1.78 |
| Currently Uninsured | -0.921*** | 0.398*** | -0.905*** | 0.405*** | -1.021*** | 0.360*** | -1.010*** | 0.364*** |
| Married | -0.027 | 0.973 | -0.012 | 0.988 | -0.062 | 0.940 | -0.048 | 0.953 |
| Spanish | 0.293* | 1.341* | 0.312** | 1.365** | 0.205 | 1.227 | 0.235 | 1.265 |
| Mexican Origin | 0.014 | 1.015 | -0.01 | 0.99 | 0.055 | 1.056 | 0.037 | 1.037 |
| Skin Color | -0.017 | 0.983 | -0.006 | 0.994 | -0.033 | 0.968 | -0.022 | 0.978 |
| Household primary care visits | 0.035** | 1.036** | 0.036** | 1.036** | 0.018 | 1.018 | 0.018 | 1.018 |
| Adjusted R-squared | 0.0573 |  | 0.06 |  | 0.0573 |  | 0.0593 |  |

*Notes:* *** p<0.01, ** p<0.05, * p<0.1, State fixed effects using complex survey weights. ^+^ Skin Color (1=Very Light, 2=Light, 3=Medium, 4=Dark, 5=Very Dark)
